# Communication strategies to encourage child participation in an oral health promotion session: An exemplar video observational study

**DOI:** 10.1111/hex.13219

**Published:** 2021-02-19

**Authors:** Siyang Yuan, Gerry Humphris, Lorna M. D. Macpherson, Alastair Ross, Ruth Freeman

**Affiliations:** ^1^ School of Dentistry University of Dundee Dundee UK; ^2^ Health Psychology School of Medicine University of St Andrews St Andrews UK; ^3^ Dental School University of Glasgow Glasgow UK; ^4^ Dental Public Health NHS Tayside Dundee UK

**Keywords:** child participation, communication behaviour, oral health promotion, triadic communication, video observation

## Abstract

**Background:**

The oral health promotion sessions for young children and parents in a clinical setting pose challenges to the dental team.

**Aim:**

To apply PaeD‐TrICS (Paediatric dental triadic interaction coding scheme) to investigate the interaction of child, parent and dental nurse and determine the effect of nurse and parental behaviours on child participation within an oral health promotion session.

**Method:**

A video observational study was applied. The sample consisted of a dental nurse and 22 children aged 2‐5 years in a general dental practice in Scotland. Behaviours were catalogued with time stamps using PaeD‐TrICS. Analysis of behavioural sequences with child participation as the dependent variable was conducted using multilevel modelling.

**Results:**

Children varied significantly in their participation rate. The statistical model explained 28% of the variance. The older the child and longer consultations significantly increased child participation. Both nurse and parental behaviour had immediate influence on child participation. Parental facilitation had a strong moderating effect on the influence of the nurse on child participation.

**Conclusions:**

Child participation was dependent on nurse and parent encouragement signalling an important triadic communication process. The coding scheme and analysis illustrates an important tool to investigate these advisory sessions designed for delivering tailored messages to young children and parents.

**Patient or Public Contribution:**

The dental staff, child patients and their parents were involved closely in the conduct and procedures of the present study.

## 1 BACKGROUND

Child participation plays a pivotal role when children attend for health‐care consultations.^1^ The importance of adopting a child‐centred approach has been advocated by the United Nations Convention on the Rights of the Child and the British Medical Association.^2^, ^3^ These policy documents have stated that every child has the right to express their views, and to be involved in communication and decision‐making process to increase the quality of care provision.^2^, ^3^ More recent evidence points to the benefits accrued to the child in the health‐care setting. These include improved parental and child satisfaction with care,^4^ increased adherence with recommended treatment options,^5^ and better health outcomes.^6^

One of the prerequisites for child participation in health‐care encounters is effective communication.^6^ Despite the evidence of improved health‐care quality for children, studies suggest that child participation and engagement in clinical settings remains low.^6^, ^7^, ^8^ It indicates that children are given less opportunities to be included as an active participant with parent and clinician (triadic interaction) in the evaluation and planning of their health care.^7^ The reasons for this lack of inclusion are believed to be complex.^9^, ^10^ First, it has almost been axiomatic over the decades that children are unable to contribute reliably to discussions about their symptoms and conditions due to their limited linguistic, cognitive and psychosocial abilities. This process is made more difficult by the complexity of managing the communication process with inclusion of three participants (ie the child patient, the parent and the health‐care provider).^10^ Second, it is not clear whether parental presence will encourage or restrict child participation in the health‐care consultations. Some research suggests parents tend to take over the turns that doctors provided for children as the parent played an “executive” role during the consultation to protect their children's welfare.^6^, ^7^, ^10^ On the contrary, other studies highlight the key role of parents in encouraging their children's participation in the clinical setting.^11^, ^12^ Furthermore, previous studies show that health‐care providers struggle to address child‐centred care issues, as on one hand, they want to involve children in their care; however, on the other hand, they are ambivalent about the level of child's involvement. This is because children may have varying preferred levels of participation based on the differing individual needs and contextual factors.^12^, ^13^

Much of this research has been conducted in the medical setting. Communicating with children in primary dental care is potentially more complex as there is a prerogative to provide appropriate information to the child for prevention and treatment as well as preparing them for dental procedures. It is recognized that the approach in which the dental professional provides this information is crucial to the young child's understanding and their parents’ assistance to enable their children to accept dental treatments.^9^, ^14^, ^15^ It is also dependent on the age of child as to how much they can be involved in the process of decision making. However, the focus of dental studies, which has examined such interactions, has concentrated on theory‐based exploration or communication and participation with little knowledge about how parents intervene and facilitate their young children's active participation during the encounter.^12^ To date, there is lack of evidence on what adult communication strategies and when these practices may encourage children's participation in the dental consultations. Moreover, evidence is equivocal regarding the effectiveness of paediatric communication strategies considering children's developmental trajectories.^12^ In order to examine the interaction, we need a valid and reliable means of measuring communication behaviours between clinician, child and parent.

The present study is conducted in a routine oral health promotion service as part of Scotland's *Childsmile* child oral health programme.^16^ In this programme, parents are encouraged to take their children to visit the dentist twice a year from the child's age of 2 years. Each dental appointment consists of fluoride varnish application, oral hygiene instruction, and advice on sugar consumption/healthy snacking and fluoride use. The participating children are video recorded with their parents in their routine Childsmile appointment with the fluoride varnish application (FVA) provided by dental health professionals. This results in the PaeD‐TrICS communication coding scheme as part of the present study. The coding scheme is developed to catalogue the communication behaviours between the dental professional, the child and the parent (triad) during the oral health promotion appointment, which includes the oral health advice and a fluoride varnish application.^17^ The use of PaeD‐TrICS may assist in unravelling the complexity of the triadic interaction and specifically the role of parent in facilitating child participation. To test the viability of this approach, a feasibility study using an exemplar case is conducted to test a quantitative methodology for analysing the detailed coded communication behaviours with an emphasis on sequencing of the three key “actors” in the dental practice. Therefore, the aims of this study are as follows: (a) to apply the new coding scheme (PaeD‐TrICS) to investigate the interaction of child, parent and dental professional; (b) to determine the effect of dental professional and parental communication behaviours on child participation within an oral health promotion session; and (c) to investigate the role of parental communication upon child participation.

## 2 METHODS

The study has adopted STROBE guidelines to report our methods.

### 2.1 Study design

A cross‐sectional observational study design is employed using a quantitative behavioural sequence approach to catalogue video recordings of oral health promotion sessions. This methodological approach treats all coded discrete behaviours as a stream of events over time in a consultation. The stream of behavioural elements from each of the participants (in this case three persons) enables cross‐lagged sequential associations to be estimated and investigated in detail.

### 2.2 Participants and setting

As part of the BEHAVE2 feasibility study, we used convenience sampling to recruit four general dental practices (five dental professionals, with 44 parental‐child dyads) working in the East of Scotland. The extended duty dental nurse (EDDN), reported here, was chosen since she worked in a general dental practice located in a socially deprived area, had over 10 years of qualified experience as a dental nurse and over 5 years of experience as a qualified EDDN. As 22 of the parent‐child pairs were recruited from her practice, we therefore further investigated her video‐recorded interactions with her child patients and parents as an exemplar study.

Child patients are eligible for study inclusion if the child is aged 2‐5 years, English spoken and with no developmental impairment. During May‐July of 2017, 28 child‐parent pairs were approached through letters with participant information sheet posted by the participating dental practice 2 weeks prior to their dental appointment. Twenty‐two consecutively attending children aged 2‐5 years and their accompanying parents for Childsmile dental appointments are recruited. Although children have rights to refuse to attend the study as part of the ethical considerations, no child or parent refused to take part. The accompanying parent is requested to provide written informed consent before participating in the study. In addition, children with siblings (*n* = 6) who attend jointly for the Childsmile dental appointment are excluded to ensure homogeneity for the interactional analysis.

### 2.3 Procedures

A small‐sized digital camera (Canon HD Camcorder LEGRIA HF R76) is placed approximately 2 m on the tripod from the participants. The operation of the recording system is hand‐held by the researcher (SY) to pick up the key features of the interaction. Twenty‐two video recordings of Childsmile dental appointments are collected. They allow direct observations of the EDDN’s communication strategies when interacting and providing preventive dental care (ie oral health advice and fluoride varnish application) to child patients with their parents. Children's age, gender and whether this is their first time to receive FVA are obtained.

#### 2.3.1 Coding scheme to assess communication: PaeD‐TrICS

The observable communication behaviours are catalogued with time stamps using the newly developed Paediatric Dental Triadic Interaction Coding Scheme (PaeD‐TrICS) by the researcher (SY). PaeD‐TrICS is developed to catalogue and define the interaction between dental professionals, parents and children in a clinical setting.^17^ This new coding scheme contains three components with 45 mutually exclusive verbal and non‐verbal behaviours exhibited by one of the three participants: that is the dental professional, the parent or the child. This comprises of a series of communication behaviours that dental professionals commonly used in managing child dental anxiety such as “TSD (tell‐show‐do) talk”, “reassurance” (eg “It's easy‐peasy”), “offer for alternative task” (eg “How about you sit on mummy's knees when having banana toothpaste?”) and those for encouraging child participation such as “praise” (eg “you are a super star”), “reward (stickers)” and “dentally engaging talk” (eg “Okay, sweetheart, tell me how many times a day you brush your teeth”).

Young children tend to have limited frequency of verbal participation within the triadic communication as observed in initial field visits. They mainly exhibit verbal responses to adult “actors” inviting questions for their contribution. The child verbal behaviours are simply categorized as “speech yes”, “speech no”, “speech other”, “laugh”, “cry” and “dental answer” when the EDDN engages them in the oral health promotion conversation using “dentally engaging talk”. The reliability of intra‐ and inter‐examiner observation is assessed (Cohen's Kappa) and found to be 0.95 and 0.83 respectively.^17^

#### 2.3.2 Recording coding communication behaviours

The coded triadic communication behaviours are recorded by bespoke designed behaviour coding software Observer XT 10.5 (Noldus Information Technology), which captures and records the details of each observable communication behaviour (utterances and/or actions) in terms of its speaker, time stamp (timing), duration (only for verbal behaviours) and occurrence (frequency).

### 2.4 Outcome measures

In this study, we only take consideration of verbal behaviours in this sequential behavioural analysis given its specific characteristics of duration.

#### 2.4.1 Child participation

Child participation is observed as their talking and speaking with the EDDN. This indicates their participation within the conversation with adult speakers. For example, children's “speech yes”, “speech other” and “dental answer” (ie child patient provided correct simple answers to EDDN’s tailored child friendly oral health‐related questions such as “how many times a day do you brush your teeth?”) are regarded as an indication of their participation, whereas their “speech no” is treated as a measure of their expressed disinterest or refusal for further participation (Table 1).

**TABLE 1:** Examples of coded communication behaviours^17^

Communication behaviour	Operational definition	Examples
Nurse behaviours		
Social talk	Non‐dentally related talk	*Hi Jo, how are you? How's school?*
Joke	Nurse makes joke/humour on the child that may include a laughter	*I can see your chatterbox tongue*
Praise	Nurse makes positive comment on child's behaviour or attitude	*You are a super star!*
Reward (stickers)	Nurse promises/gives child a reward, often dependent on behaviour	*You will get a sticker after you have banana toothpaste, how does that sound?*
Dental engaging talk	Any talks nurse uses to get child engaged in the oral health‐related talk/treatment	*So, after you brush your teeth before you go to bed, do you have anything else to eat or drink or you just go straight to bed?*
Parent behaviours		
Parental facilitation	Parent helps nurse or child to convey information for easier understanding to the third party	*After the nurse asked the child whether he had anything to drink before bedtime, Mum joined the conversation, ‘What sometimes happens? (silence in 2 seconds) You have a sneaky drink (Mum lowered her voice like a whisper to the child)’*
Joke	Parent makes joke/humour on the child that may include a laughter	*When the nurse asked the child whether he had anything to eat or drink at bedtime, Mum smiled and winked at the child, ‘Hmm…this is the sticky point, isn't it?’*
Praise	Parent makes positive comment on child's behaviour or attitude	*You did a great job, sweetheart. Well done!*
Child behaviours		
Speech yes	Child says ‘yes’ or expresses agreement	*Uh‐huh*
Speech no	Child says ‘no’	*No*
Speech other	Child says any other utterances except for ‘yes’ and ‘no’	*‘I bumped my knees at the nursery yesterday’. (child told the nurse what happened to his school yesterday)*
Dental answer	Child says anything to reply DP’s oral health‐related question	*When mum facilitated the nurse's question, the child answered, ‘Emm…bedtime’*

#### 2.4.2 Adult engagement behaviours

In this exemplar study, we select five most frequent dental nurse's observed behaviours as approaches to engaging child patients in the interaction. These verbal behaviours include “dentally engaging talk”, “praise”, “reward (sticker)”, “social talk” and “joking”. Parents’ talk to enable their children's participation is less diverse than the dental nurse's communication behaviours. Parents’ behaviours observed are predominately “parental facilitation”, “praise” and “joking” (Table 1 presents examples of observed verbatim expressions and any non‐verbal behaviour or contextual information for each behavioural code entered into the detailed analysis).

### 2.5 Data analysis

Our theoretical framework is that adult behaviours (nurse and/or parent) might have an important part to play in child participation in the dental consultation.^8^ Based on this framework, we believe that the nurse's child‐centred engagement strategies (such as “praise” or “dentally engaging talk”) or parental verbal behaviour (eg “joking” or “parental facilitation” to reinforce nurse's communication) may affect positively the child's participation within the conversation. We created a behaviour “chain” by selecting the nurse's five most frequently used verbal behaviours and three parental behaviours followed by child's verbal response (ie “child participation”) in the final analysis. Such a relatively simple, but clearly defined, behavioural “chain” is considered as a behaviour sequence in our analysis.

**FIGURE 1:**
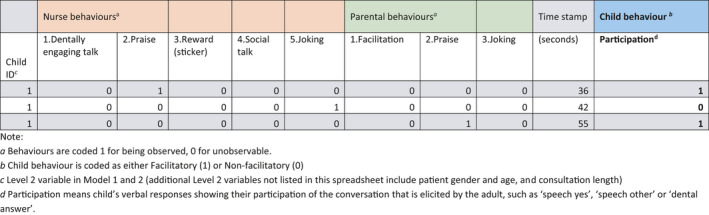
Spreadsheet of the first three rows of example data file to illustrate formatting in preparation for linear modelling. ^a^Behaviours are coded 1 for being observed, 0 for unobservable. ^b^Child behaviour is coded as either Facilitatory (1) or Non‐facilitatory (0). ^c^Level 2 variable in Model 1 and 2 (additional Level 2 variables not listed in this spreadsheet include patient gender and age, and consultation length). ^d^Participation means child's verbal responses showing their participation of the conversation that is elicited by the adult, such as “speech yes”, “speech other” or “dental answer”

The pairings or “chains” were collected as the sequences of all the selected behaviours with time stamp were uploaded to STATA/IC™ v14. The *xmelogit* procedure using maximum likelihood via adaptive Gaussian quadrature estimation methods was employed on the categorical child behaviour dichotomous variable. To illustrate the coded data set formatted for analysis within STATA an example is presented (Figure 1). The analysis of behavioural sequences with child participation as dependent variable (scored 0 for no child participation and 1 for child participation) was conducted using multilevel modelling with nurse and parental behaviours as independent explanatory variables at Level 1, and Child Identification number at Level 2. Child age, gender, behavioural time stamp and length of consultation were treated as covariates. The inclusion of the time stamp we believe is vital to our understanding of the processes in a consultation. For example, a close inspection of the adult “turns” at the various phases of the consultation enables an appreciation of the importance of the behaviour on the child participation at various phases during the dental visit.

## 3 RESULTS

### 3.1 Consultation structure

A typical structure of the Childsmile consultation provided by the EDDN includes a social talk at the beginning, oral health instruction with the family (both child and parent) involving a toothbrushing demonstration using a tooth model and a toothbrush, and a fluoride varnish application at the end.

### 3.2 Characteristics of participants

Twenty‐two child‐parent pair participate in the study (*N* = 22). Children's age ranges from 26 to 64 months with a mean age of 45.75 months and the median being 47.5 months. Thirty‐six percent of children are girls.

### 3.3 Description of variables

Summary statistics of variables included in levels 1 and 2 are analysed (Table 2). The frequency of child participation is 22.8% of the complete 2538 behavioural sequences. Five dental nurse and three parental behaviours are found to describe 97% of the recorded entire coded behavioural repertoire of the two adult “actors”. Over 27% of all recorded behaviours are exhibited by the dental nurse in the various verbal utterances containing advice, information, questions and recommendations. Likewise, the parent presents a similar frequency level (28%) in the form of facilitation. The average duration of a behavioural sequence was 2.7 seconds ranging from just under a tenth to a maximum of 46 seconds demonstrating considerable range. The average length of consultations including FVA and oral health advice is 19 minutes (range 10‐29).

**TABLE 2:** Basic aggregate statistics across consultations and “actor” behaviours according to variable type

Outcome variable at level 1 (*n* = 2578)		
Child participation	22.8% (587)	
Explanatory variable		(min‐max)
Level 1 (behavioural sequence, *n* = 2578)		
Dentist behaviour		
Talk^b^	27.1% (299)	
Praise^b^	10.2% (262)	
Social^b^	10.3% (265)	
Joke^b^	7.4% (190)	
Reward^b^	7.7% (199)	
Parent behaviour		
Facilitate^b^	27.9% (718)	
Praise^b^	2.3% (58)	
Joke^b^	4.1% (106)	
Behaviour duration^a^ (s)	2.27 (SD = 2.43)	(0.08‐45.68)
Level 2 Consultations (*n* = 22)		
Patient age^a^ (mths)	47.32 (SD = 13.05)	(26‐64)
Length of consultation^a^ (s)	1121.71 (SD = 291.12)	(588‐1755.5)
Patient sex^b^ (reference: girl)	32% (7)	

^a^ Continuous variables are presented with means, standard deviations (SDs), and minimum and maximum values (in brackets).

^b^ Dichotomous variables are presented with percentages and absolute figures (in brackets). Note percentages referring to behaviours denote the presence (not absence).

### 3.4 Prediction of child participation

Table 3 shows the multilevel, multivariate logistic regression results for child participation including the level 1 and level 2 predictors. The strongest predictive model is Model 2 that includes both EDDN and parental behaviours controlling for length of these behaviours, consultation duration, gender and age of child. Model 1 is instructive as it demonstrates that concentrating on the dental nurse's behaviour and broad contextual variables such as length of consultation, and essential child demographics (age and gender) explains substantial variance beyond the simple information contained in the identity of the child (ie null model). The detailed inspection of regression coefficients in Model 1 is suspended as there are substantial changes in these coefficients, including both magnitude and direction when including parental behaviour. Moreover, the Model 2 significantly improves prediction of child participation over Model 1 as confirmed by the log‐likelihood test (Chi Square = 195.3, *P* = .0001). However, a consistent finding, across both Models, is found with dental nurse's “Talk” (ie “dentally engaging talk”) behaviour. A detailed interpretation of the parameter estimates in Model 2 reveals dental nurse behaviour increases the chances of child participation by a factor of nearly 11, whereas this prediction of participation is raised by a factor of 6 times when parental facilitation is observed. Furthermore, the EDDN’s social talk and rewards tend to increase child participation. As the consultation progresses, however, any parental or EDDN behaviour that was exhibited tend to reduce the child's participation, as confirmed by the entry of the “Behaviour time” variable into this final Model 2.

**TABLE 3:** Multilevel regression results of various Models showing parameter estimates (Maximum Likelihood) across Levels 1 and 2, 95%CIs and *P* levels, including random effects, log‐likelihood ratio tests and intra‐class correlation

	Null model	Model 1			Model 2		
		OR	95% CI	*P*	OR	95% CI	*P*
*Fixed effects*							
Level 1							
Nurse behaviour							
Talk^b^		2.37	1.83, 3.05	.0001***	10.85	3.81, 30.9	.0001***
Praise^b^		0.40	0.25, 0.64	.0001***	1.79	0.59, 5.49	.30
Social^b^		0.96	0.67, 1.40	.86	4.37	1.49, 12.8	.007**
Joke^b^		0.46	0.26, 0.80	.007**	2.06	0.64, 6.62	.22
Reward^b^		1.24	0.81, 1.89	.32	5.68	1.89, 17.1	.002**
Parent behaviour							
Facilitate^b^					6.38	2.23, 18.2	.001***
Praise^b^					1.32	0.27, 6.48	.74
Joke^b^					0.50	0.10, 2.37	.38
Behaviour time^a^					0.99	0.99, 0.99	.0001***
Level 2 Consultations (*n* = 22)							
Patient age (mths)		1.04	1.01, 1.08	.009**	1.04	1.01, 1.08	.008**
Length^a^		1.002	1.000, 1.003	.037*	1.001	1.000,1.003	.048*
Patient sex^c^		1.79	0.76, 4.21	.18	1.76	0.74, 4.23	.20
*Random effects (intercept)*							
Level 2 variance (95% CI)	1.14 (0.81,1.61)	0.86 (0.61, 1.22)			0.88 (0.62,1.25)		
Level 2 ICC	28.4%						
Log likelihood	−1228.9	−1142.9			−1115.1		
*LR^1^* test	*Χ^2^* = 308.34, *P* = .0001	*Χ^2^* = 153.76, *P* = .0001			*Χ^2^* = 177.16, *P* = .0001		
*LR^2^* test	n/a	*Χ^2^* = 189.9, *P* = .0001			*Χ^2^* = 195.3, *P* = .0001		

Note

*LR^1^* test: Likelihood ratio test comparing the mixed effects logistic model to standard logistic model, *LR^2^* test: likelihood ratio test for model improvement. *denotes *P* < .05, **denotes *P* < .01, ***denotes *P* < .001.

Abbreviation: ICC, Intra‐class correlation.

^a^ Grand mean centred (secs).

^b^ Coded: not observed 0, Behaviour type observed 1.

^c^ Coded: female 0, male 1.

As a check of computational efficiency, all models presented in Table 3 are rerun with GLLAMM (Generalized Linear Latent And Mixed Models) and are found, reassuringly, to give virtually identical parameter estimates (results retained).^18^ The *xmelogit* procedure within STATA is the preferred routine for estimation due to speedier processing and selected for presentation purposes.

## 4 DISCUSSION

Child participation is critical for providing child‐centred care.^2^, ^3^, ^15^ The medical literature suggests young children have little participation in the paediatric consultations and parents mostly act as their safeguard to exchange information and make decisions.^7^, ^10^ However, there is a paucity of empirical research in dentistry with respect to child participation in dental settings. Moreover, the parental presence in the dental surgery has been a heated debate for decades with diverging opinions. Our study indicates that the parent may serve as a linchpin to facilitate the interaction between the dental nurse and the young child patient in an oral health‐promoting consultation.

The first objective of this exemplar study has been met—namely the successful application of PaeD‐TrICS to record the triadic interactions. The feasibility challenge of consenting staff and patient/carer was overcome. None of the participants regarded the observational procedure invasive and all allowed the researchers to record video and report verbatim utterances with confidentiality assurances. The coding is completed from the video recordings, and the resulting numerical data sets are converted appropriately into a format that enables longitudinal intensive sequential analysis. Our focus is the child's participation in the consultation. Researchers have the opportunity to identify any key behaviour that theoretically is interesting and generates a need to investigate closely.

The second objective is achieved that is to obtain estimates of the associations of dental nurse behaviours on child participation and likewise of parental behaviours on their child's involvement in the consultation. With the proviso that the detailed analysis presented only reflects the set of associations for a single EDDN, it is found that the influence of staff and parental behaviour is marked. When the dental nurse's behaviours are entered initially (without inclusion of the parents’ behaviour), some clear behavioural effects of the nurse influence on child participation are evident. Nurse talking about dental matters using questioning skills (ie “dentally engaging talk”) strongly engage the child. “Praise” predicts less participation by the child whereas making a joke increases child participation. The use of praise is a frequent behaviour exhibited by this nurse and will not discriminate to stimulating the child to show participatory behaviour.

It has been noted that parent's behaviours may inadvertently encourage or indeed prevent their children's participation during the nurse's interaction with the child. Nevertheless, these events were double checked by the two coders. These events were regarded as minimal in the consultations as the dental nurse acted as a conduit between parent and child in the triadic interaction. This was observed as the parents apparently remaining in the background and leaving their child to interact with the nurse. Parents only intervene when they regarded (“see”) their child having difficulty to respond to the nurse's questions. A typical case that appeared in our observation of such a verbal sequence is that usually after the dental nurse invited the child to respond to her questions, the parent would wait for 2‐3 seconds before facilitating (“parental facilitation” behaviour) further interaction. Therefore, it may be considered that the effect of “parental facilitation” is to assist the child to respond and related to the EDDN, that is, the formation of the triadic alliance.

However, a very different picture is obtained when introducing the parental behaviours into the model (see Model 2 in Table 3). The third objective to investigate the role of parental communicative behaviours presents a clear pattern with this dental nurse. Again, the dental talk behaviour is consistent in predicting child participation. It is interesting though that the inclusion of the parental behaviours in the final model shows that nurses’ praise and joking behaviours are neutral in predicting child participation. The nurse using “social talk” and “rewards” has improved the child's participation with a similar strong effect of the parent facilitating the nurse's interaction strategies and advice with the child. One particular observation noted is the key role of the trusting relationship between the parent and the dental nurse. Parents remain in the backstage to give space for the older child (normally aged 4‐5 years) to interact with the nurse. They only step in to play as the “translator” for the child when they sense the obstacles during the interaction between the child and the nurse. Such trusting relationship between the two adults enables a treatment working alliance to make collective effort to support child participation. In other words, the working alliance between the dental nurse and the parent is the prerequisite for a successful facilitation to engage the child in the dental consultation. This has been echoed in the paediatric literature.^10^, ^11^

Of note is the association of the time of the behaviour (either nurse or parent) and child participation. The more extended the duration of the consultation, the less likely the communicative behaviours of the adults are exhibited to show participation of the child. An important implication may be that the initial communication with the child in the dental visit is crucial with less emphasis on subsequent behaviours later in the consultation to influence child participation. We believe this is the first empirical demonstration of such an effect, although we highlight that this is only shown with a single member of staff.

There are some important general effects reflected in the Level 2 covariates. The older the child and the longer the consultation, the more extensive child participation. Some evidence from the BEHAVE study that investigated 270 nurse‐child interactions for fluoride varnish applications in kindergarten settings in Scotland, had shown that a longer consultation was associated with poorer cooperation in receipt of a fluoride varnish.^19^ Consistent with the BEHAVE study, it was found that age of child improved cooperation. Of note is the child participants in BEHAVE study aged 3‐5 years, which is similar to the age group of children we examined. Cooperation and participation of the young child patient are not identical behaviours; however, they are linked. In the context of this general dental practice, the nurse spent longer with the child and generated possibly a greater rapport to increase child participation. In the BEHAVE study that was conducted in a community setting (ie kindergarten), encouraging a reluctant child to receive a varnish may extend the consultation without achieving easily a successful procedural outcome by seeking the child's cooperation to receive the fluoride application.

We believe that the next stage of future work with this behavioural sequencing approach should focus on using a three level multilevel model. The extra level would refer to additional members of staff. We have already reported a preliminary study comparing dentists and dental nurses.^8^ The major differences in frequencies of specific professional behaviours were described; however, the influence of these behaviours on the child was not investigated in detail as the sample sizes of children and staff were too modest. To enable a more generalizable and therefore informative description of the sequential associations between parents, staff members and the child, a suitable range of staff are required with a minimum sample size of twenty with each offering to record interactions with twenty children. The number of consultations recorded would sum to 400 and the estimated number of behaviours per consultation, as we have demonstrated, would average approximately 250. We believe the investment in preparing such a database would be handsomely returned in providing for the first time a catalogue of findings to assist dental staff with a scientific basis for their communication skills with this particularly young age group of patients and thereby assist with the development of training programmes. In order to develop child‐centred communication approaches it is important that EDDNs recognize that preschool children's shyness is in accordance with the phase of their psychological development and for EDDNs to have a cogent understanding of what the dental treatment experience means to the child at the stage and phase of their psychological development.^20^

We have already alerted the reader to the limitation of interpreting these results with a single member of staff and only 22 child‐parent pairs. We admit that the exploratory nature of the exemplar study with such a small sample size restricted the generalizability of the study. However, more importantly, the findings suggest parents have significant influence on the nurse‐child interaction. In terms of the statistical aspect, our model is based on a single sequential lag. That is an adult behaviour is expressed and the next instance of child behaviour is noted. Our selection of the immediate effect of an adult behavioural element (lag 1) on the child seems an obvious choice. Further lags are not investigated. The complexity of including additional lags into this sequential approach is beyond the scope of this model but the investigators are well aware that additional models can be applied.

A recent investigation conducted in Hong Kong reported the multi‐party communication process of dental caries prevention from 77 video recordings of the dentist, dental assistant (acting as translator), child patient and their carer in consultation.^21^ This research group employed visual text analysis and conversational analysis to illustrate their findings. These two approaches are sophisticated qualitative methodologies that contribute to our understanding of the complexity of such clinical interactions. Our approach is more quantitative and has the advantage of identifying key factors using conventional hypothesis testing and specification of what might be regarded as generalizable samples. The report we present is an exemplar of what can be achieved with a sufficient pool of practices, staff and patients. A limitation, in addition, of our report is that the practice followed their own interpretation of the *Childsmile* programme. Further work would be beneficial to provide a more comprehensive description of the communication processes adopted by staff and the influence of the parent/carer in the fluoride varnish application and dental health education practice.

## 5 CONCLUSION

This study has demonstrated, we believe, a powerful methodology that has been lacking in previous inspections of dental staff and child behaviours. The inclusion of an exquisitely detailed coding scheme, and the parental behaviour, as well as that of the dental professional, enables a tripartite analysis that provides an important additional approach to unlock the dynamics of the complex communication process that exists in the dental health consultation.

## AUTHORS’ CONTRIBUTION

SY involved in study conception and design, acquisition of data, analysis and interpretation of data, drafting of the manuscript and critical revision. GH involved in conception and design of the study, analysis and interpretation of data, drafting of the manuscript and critical revision. LMDM involved in conception and design of the study. AR involved in conception and design of the study, acquisition of data. RF involved in funding receiver, study conception and design, drafting the manuscript and critical revision.

## ETHICAL APPROVAL

The study was reviewed and given an ethical approval by the East of Scotland Research Ethics Service (Ref: 16/ES/0081). The present project adhered to guidelines of the Declaration of Helsinki and the participating dental health professionals and parents have provided their written informed consent to participate this study prior to the Childsmile dental appointments.

## Data Availability

Author elects to not share data. Research data are not shared. The data set generated and analysed is not publicly available as it is required by the ethical approval (Ref: 16/ES/0081) granted by the East of Scotland Research Ethics Service that the data set contains video data that could potentially identify participants and/or health‐care facilities.
